# Suppression of microRNA-16 protects against acute myocardial infarction by reversing beta2-adrenergic receptor down-regulation in rats

**DOI:** 10.18632/oncotarget.15391

**Published:** 2017-02-16

**Authors:** Jiaqi Liu, Fei Sun, Yuying Wang, Wanqi Yang, Hongwen Xiao, Yue Zhang, Renzhong Lu, Haixia Zhu, Yuting Zhuang, Zhenwei Pan, Zhiguo Wang, Zhimin Du, Yanjie Lu

**Affiliations:** ^1^ Department of Pharmacology State-Province Key Laboratories of Biomedicine-Pharmaceutics of China, Key Laboratory of Cardiovascular Research, Ministry of Education, College of Pharmacy, Harbin Medical University, Harbin, P.R. China; ^2^ Institute of Clinical Pharmacology of the Second Affiliated Hospital Key Laboratory of Drug Research, Heilongjiang Higher Education Institutions, Harbin Medical University, Harbin, P.R. China; ^3^ Northern Translational Medicine Research and Cooperation Center, Heilongjiang Academy of Medical Sciences, Harbin Medical University, Harbin, P.R. China

**Keywords:** acute myocardial infarction, miR-16, beta2-adrenergic receptor, apoptosis

## Abstract

microRNA-16 (miR-16) has been shown to be up-regulated in ischemic heart. Beta2-adrenoreceptor (β_2_-AR) exerts cardioprotective property in ischemic injury. This study aims to determine the effect of miR-16 in cardiac injury in rats and the possible involvement of β_2_-AR in this process. Acute myocardial infarction (AMI) model in rats was induced by ligation of left coronary artery. Neonatal rat ventricular cells (NRVCs) were cultured *in vitro* tests. The cardiomyocyte model of oxidative injury was mimicked by hydrogen peroxide. The expression of miR-16 was obviously up-regulated and β_2_-AR was remarkably down-regulated in both AMI rats and NRVCs under oxidative stress. miR-16 over-expression in NRVCs reduced cell viability and increased apoptosis. Conversely, inhibition of endogenous miR-16 with its specific inhibitor reversed these changes. Over-expression of miR-16 using an miR-16 lentivirus in AMI rats markedly increased cardiac infarct area, lactate dehydrogenase and creatine kinase activity, and exacerbated cardiac dysfunction. Lentivirus-mediated knockdown of miR-16 alleviated acute cardiac injury. Moreover, miR-16 over-expression significantly suppressed β_2_-AR protein expression in both cultured NRVCs and AMI rats, while inhibition of miR-16 displayed opposite effect on β_2_-AR protein expression. Luciferase assay confirmed that miR-16 could directly target the 3′untranslated region of β_2_-AR mRNA. miR-16 is detrimental to the infarct heart and suppression of miR-16 protects rat hearts from ischemic injury via up-regulating of β_2_-AR by binding to the 3′untranslated region of β_2_-AR gene. This study indicates that targeting miR-16/β_2_-AR axis may be a promising strategy for ischemic heart disease.

## INTRODUCTION

Acute myocardial infarction (AMI), a major cause of sudden death, is a life-threatening disease that is characterized by hypoxia and death of cardiac myocytes due to the occlusion of coronary vessels [1]. For those who survived, the heart may undergo post-infarct remodeling such as interstitial fibrosis and cardiac hypertrophy that may lead to heart failure [2, 3]. To improve the prognosis of AMI, it is essential to attenuate the loss of cardiomyocytes in early phase. Although great efforts have been devoted to the development of novel cardioprotective agents, the progress appears to be far from satisfying. There remains an urgent need for the elucidation of the molecular mechanisms of AMI and the invention of new therapeutic strategy.

Beta1-adrenoreceptor (β_1_-AR) and beta2-adrenoreceptor (β_2_-AR) are the main isoforms of beta-adrenoceptor in myocardium, which exert differential effects on the survival of cardiac myocytes. β_1_-AR mediates the cardiotoxic effect of norepinephrine via cyclic adenosine monophosphate (cAMP)-dependent signaling pathway, while β_2_-AR protects cardiac myocytes via a Gi-mediated mechanism [4, 5]. Up-regulation of β_2_-AR by arformoterol mitigates cardiac ischemia injury through the activation of NO synthase [6]. Knockout of β_2_-AR exacerbates doxorubicin induced cardiac toxicity via the suppression of survival kinases and enhancement of intracellular calcium [7]. Furthermore, β_2_-AR activation has been implicated in the preservation of cardiac function in a rat model of ischemic heart failure by inhibiting apoptosis and cardiac remodeling [8–10].

MicroRNAs (miRNAs) are a group of small non-coding RNAs with 21-25 nucleotides in length that negatively regulate the expression of target genes [11]. Increasing evidence indicates that miRNAs play important regulatory roles in the pathogenesis of various cardiac diseases [12–14]. Over-expression of miR-320 increases cardiomyocyte death and apoptosis by targeting heat-shock protein 20 [15]. miR-210 and miR-150 protect the heart from ischemic injury by regulating cell death [16, 17]. miR-16 is initially known as a tumor suppressor as it induces cancer cell apoptosis [18, 19] and negatively regulates cellular growth and cell cycle progression [20]. Notably, miR-16 is highly expressed in cardiomyocytes [21], while its functional role in the heart remains unclear. In this study, we explored the role of miR-16 in AMI and its regulatory effect on β_2_-AR, and found that miR-16 exacerbated cardiac injury by suppressing the expression of β_2_-AR, which provides new insight into the mechanism of AMI.

## RESULTS

### Levels of miR-16 and β_2_-AR in cardiac tissues of AMI rats and neonatal rat ventricular cells (NRVCs) treated with hydrogen peroxide (H_2_O_2_)

**Figure 1:**
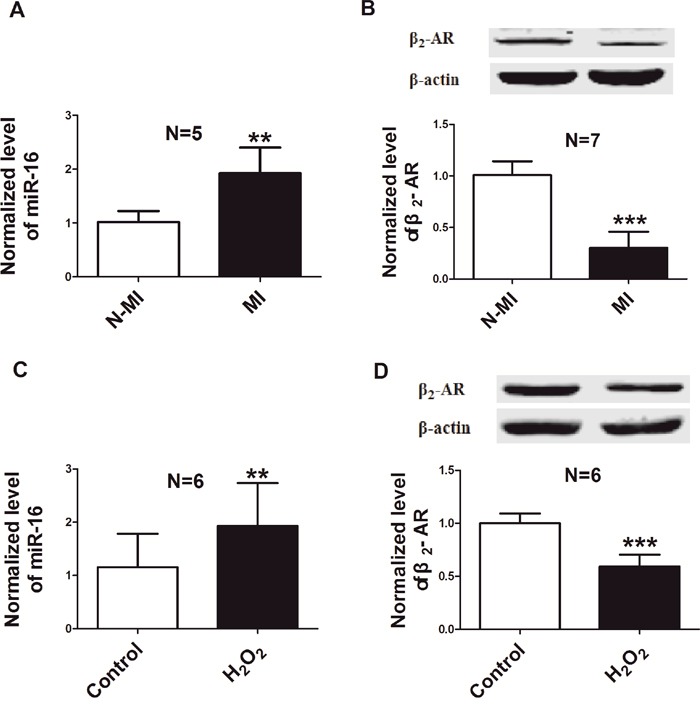
Down-regulation of β_2_-AR and up-regulation of miR-16 in rat model of AMI and in a cellular model of H_2_O_2_ damage **A**. Level of miR-16 in the infracted area 6 h after AMI, compared to non-ischaemic area of left ventricle from the same heart by real-time RT-PCR. ^*^P < 0.01 vs. N-MI. n=5. **B**. β_2_-AR protein level in the infracted area 6 h after AMI, compared with non-ischaemic area of the same heart by western blot. ^**^*P < 0.001 vs. N-MI. n=7. **C**. Level of miR-16 in NRVCs treated with 100μM H_2_O_2_ for 12 h. n=6. ^*^P < 0.01 vs. control. **D**. Expression of β_2_-AR in NRVCs treated with H_2_O_2_ (100μM). n=6. ^**^*P < 0.001 vs. control. N-MI, non myocardial infarction; MI, myocardial infarction. Data are expressed as mean ± SD.

Rat AMI model was established by permanent ligation of left anterior descending coronary artery (LAD). Levels of miR-16 and β_2_-AR were detected in the infarcted tissue of rat hearts 6 h after AMI. As illustrated in Figure 1A & 1B, the expression of miR-16 in the infract area was up-regulated and the protein level of β_2_-AR was down-regulated dramatically compared with non-ischaemic area of the same heart. Then we examined the expression of miR-16 and β_2_-AR in cellular damage model which was established by treating NRVCs with H_2_O_2_ (100 μM) for 12 h. Consistent with the observation in animal model, the level of miR-16 was increased, while the protein expression of β_2_-AR decreased in NRVCs subjected to H_2_O_2_ damage (Figure 1C & 1D).

### Effects of miR-16 on cardiomyocyte damage *in vitro*

**Figure 2:**
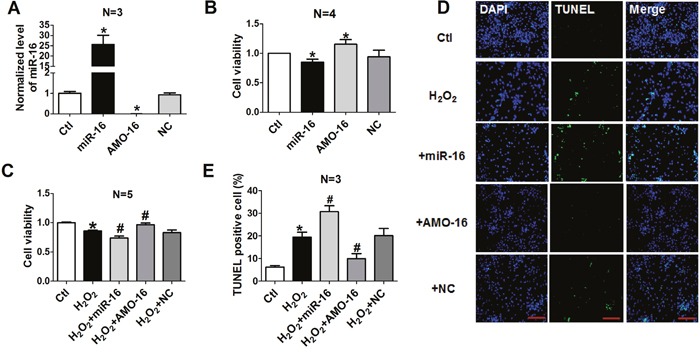
Effects of miR-16 on cardiomyocyte cell vitality and apoptosis **A**. miR-16 levels in NRVCs transfected with miR-16, AMO-16 or NC by real-time RT-PCR. *P < 0.05 vs. control.^*^P < 0.01 vs. control. n=3. **B, C**. Cell viability determined by MTT assays. NRVCs were transfected with NC, miR-16, AMO-16 for 24 h, then treated with or without H_2_O_2_ (100 μM) for 12 h. ^*^P < 0.01 vs. control. ^#^P < 0.05 vs. H_2_O_2_.^##^P < 0.01 vs. H_2_O_2_.n=5. **D**. Representative images of TUNEL staining showing apoptotic cells (stained in green). The nuclei were stained blue with DAPI. **E**. Percentage of TUNEL-positive cells. *P < 0.05 vs. control.^#^P < 0.05 vs. H_2_O_2_. Data are expressed as mean ± SD.

We then investigated whether the aberrant up-regulation of miR-16 exerts any functional influence on the viability and apoptosis of cardiomyocytes. miR-16 mimics (miR-16), its specific inhibitor (an anti-miR-16 antisense oligodeoxyribonucleotide, AMO-16) and the negative control (NC) were transfected with X-treme GENE siRNA transfection reagent into NRVCs for 48 h. The efficiency of miR-16 transfection was verified by real-time PCR (Figure 2A). miR-16 level was increased by approximately 25-fold after miR-16 transfection compared with control group, and dropped to almost non-detectable level after AMO-16 transfection. The level of miR-16 was unchanged in NC group. The role of miR-16 on NRVCs viability was evaluated by 3-[4, 5-dimethylthiazol-2-yl]-2, 5 diphenyl tetrazolium bromide (MTT) assay. We found that over-expression of miR-16 reduced, while AMO-16 increased cell viability (Figure 2B). The cell viability was significantly decreased in NRVCs treated with H_2_O_2_ (100 μM) for 12 h. Over-expression of miR-16 markedly exaggerated this detrimental change. On the contrary, down-regulation of miR-16 with AMO-16 significantly increased cell viability, whereas treatment of NC had no such effect (Figure 2C). To confirm whether the changes of cell viability were at least partially attributable to myocardial apoptosis, TUNEL assay was performed in a forthcoming experiment. TUNEL positive cells were increased in miR-16-transfected cardiac myocytes with treatment of H_2_O_2_. However, inhibition of miR-16 rescued the cellular damage induced by miR-16 (Figure 2D & 2E). These results indicate that miR-16 exacerbated cardiomyocyte injury by affecting cell viability and apoptosis *in vitro*.

### Effects of miR-16 on cardiac injury of AMI rats *in vivo*

**Figure 3:**
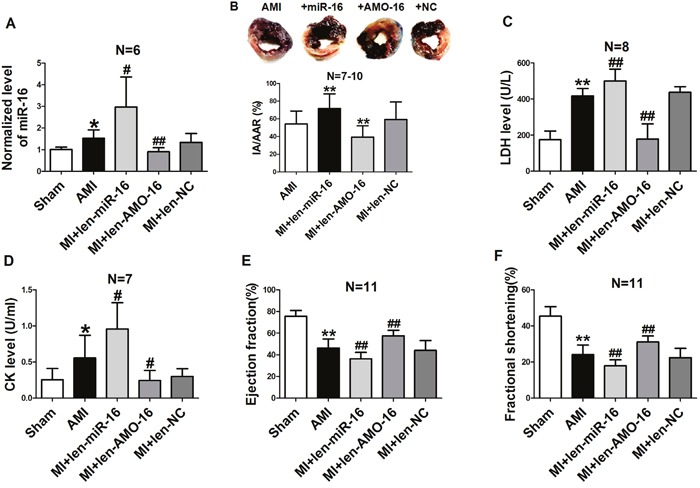
Effects of miR-16 on infarct size, cardiac function, lactate dehydrogenase (LDH) and creatine kinase (CK) in acute myocardial infarcted rats **A**. Levels of miR-16 in the AMI rat hearts treated with len-pre-miR-16, len-AMO-16 and len-NC. *P < 0.05 vs. Sham. ^#^P < 0.05 vs. AMI. ^##^P < 0.01 vs. AMI. n=6. **B**. Analysis of IA/AAR ratio. IA, infarct area; AAR, area at risk. ^*^P < 0.01 vs. AMI. n=7-10. **C**. Serum LDH level. ^*^P < 0.01 vs. Sham.^##^P < 0.01vs. AMI. n=8. **D**. Serum CK level. *P < 0.05 vs. Sham.^#^P < 0.05 vs. AMI. n=7. **E**. Ejection fraction (EF) of the hearts.^*^P < 0.01 vs. Sham.^##^P < 0.01vs. AMI. n=11. **F**. Fractional shortening (FS) of the hearts. ^*^P < 0.01 vs. Sham.^##^P < 0.01vs. AMI. n=11. Data are expressed as mean ± SD.

Since miR-16 plays an important role in H_2_O_2_-induced myocardial injury in NRVCs, we wanted to further elucidate the effect of miR-16 upon AMI *in vivo*. Thus, lentivirus vectors containing pre-miR-16 sequence (len-pre-miR-16), The AMO of miR-16 (len-AMO-16) and mismatched sequence (len-NC) were injected into the left ventricular cavity 7 days prior to AMI in rats. Our results demonstrated that the level of miR-16 was increased in AMI rat hearts administered with len-pre-miR-16 compared with the control animals, and it was decreased in the len-AMO-l6 group. Len-NC had no effect on miR-16 expression (Figure 3A). We then assessed the myocardial infarct size by Evans blue and triphenyltetrazolium chloride (TTC) staining 6 h after coronary ligation. We found that the infarct area (IA) / area at risk (AAR) ratio was larger in len-pre-miR-16 group than in AMI rats (Figure 3B), while knockdown of miR-16 with len-AMO-16 reduced IA/AAR ratio. Serum lactate dehydrogenase (LDH) and creatine kinase (CK) are important markers for acute cardiac injury. We found that miR-16 over-expression further elevated the release of serum LDH and CK in AMI rats, which len-AMO-16 treatment reduced serum LDH and CK release (Figure 3C & 3D). Moreover, the echocardiographic data indicated that len-AMO-16 dramatically improved cardiac function in AMI rats, as reflected by increasing ejection fraction (EF) and fractional shortening (FS) (Figure 3E & 3F). Compared with AMI and len-miR-16 groups, len-AMO-16 did not have significant effect on the heart rate of rats (data not shown).

### Expression of β_2_-AR is regulated by miR-16

**Figure 4:**
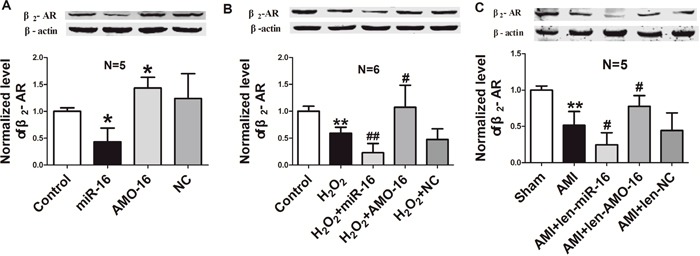
β_2_-AR expression is repressed by miR-16 in neonatal rat ventricular cells and ischemic hearts **A**. miR-16 mimics and inhibitor were transfected into NRVCs cells, and then the expression of β_2_-AR was detected by western blotting assay. *P < 0.05 vs. control. n=5. **B**. Repression of β_2_-AR protein by miR-16 induced by oxidative stress in NRVCs transfected with NC, miR-16 and AMO-16 for 24 h, then treatment with H_2_O_2_ (100 μM) for 12 h. ^*^P < 0.01 vs. control.^#^P < 0.05 vs. H_2_O_2_. ^##^P < 0.01 vs. H_2_O_2_. n=6. **C**. The protein levels of β_2_-AR in the ischaemia tissue (6 h of ischaemia) pre-treated with len-pre-miR-16, len-AMO-16 and len-NC for 7 days. ^*^P < 0.01 vs. Sham.^#^P < 0.05 vs. AMI. n=5. Data are expressed as mean ± SD.

Considering the involvement of β_2_-AR in cardiac injury [6], and the observation that the expression of β_2_-AR and miR-16 was inversely changed in both cardiac tissues of AMI model and NRVCs subjected to H_2_O_2_ damage as shown in Figure 1, we speculated that miR-16 may participate in cardiac injury by regulating the expression of β_2_-AR. Thereby, we examined the expression β_2_-AR in NRVCs following miR-16 over-expression, and found that the protein level of β_2_-AR was significantly inhibited in cultured cardimyocytes by miR-16 relative to control group. In contrast, it was obviously increased in cells transfected with AMO-16. The NC did not show any influence on expression of β_2_-AR (Figure 4A). We then examined the changes of β_2_-AR protein level in NRVCs under oxidative stress. The results showed that transfection of miR-16 remarkably decreased β_2_-AR protein expression in NRVCs in response to H_2_O_2_, and this effect was abrogated by AMO-16 (Figure 4B). Consistently, in AMI rats len-pre-miR-16 treatment profoundly caused down-regulation of β_2_-AR in cardiac tissue, which was reversed by len-AMO-16. β_2_-AR expression was not changed in len-NC group (Figure 4C).

### Validation of β_2_-AR as a target of miR-16

**Figure 5:**
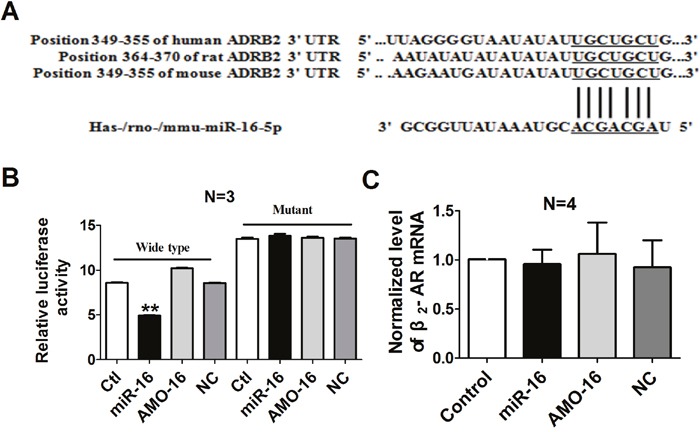
Verification of β_2_-AR as a target for miR-16 **A**. The 5′end seed region of miR-16 had complementary sites in the β_2_-AR mRNA 3′UTR. **B**. The interactions between miR-16 and its binding sites in the 3′UTR of β_2_-AR were measured by luciferase reporter gene assay in HEK293 cells. The intensity of luciferase was detected 36 h after transfection. ^*^P < 0.01 vs. control. n=3. **C**. Effects of miR-16 on the mRNA level of β_2_-AR in cultured cardiac myocytes. n=4. Data are expressed as mean ± SD.

To further identify whether a targeting relationship is existed between miR-16 and β_2_-AR, three bioinformatic programs (TargetScan, miRDB and miRanda) were used to predict the potential target genes of miR-16. The β_2_-AR-encoding mRNA contains a 3′UTR binding site for miR-16, and the complementary sequence was highly conserved among human, rat and mouse region, implying β_2_-AR might be a potential target for miR-16 (Figure 5A). Therefore, dual-luciferase reporter analysis was performed to experimentally establish β_2_-AR as a target gene for miR-16. We performed luciferase reporter activity assay in HEK293 cells with the vectors engineered to carry the 3′UTR of β_2_-AR mRNA. The data showed that over-expression of miR-16 decreased the luciferase activity of the reporter gene with wild type luc-ADRB2 3′UTR. However, transfection of miR-16 had no effect on the luciferase activity of the reporter carrying the mutated miR-16 binding site (Figure 5B). All these data support the specificity of miR-16 action on the 3′UTR of β_2_-AR mRNA. We also examined the mRNA level of β_2_-AR after miR-16 treatment, and it turned out that miR-16 produced no influence on β_2_-AR mRNA (Figure 5C).

## DISCUSSION

In the present study, we found that miR-16 was aberrantly up-regulated during cardiac injury. Over-expression of miR-16 exacerbated cardiac injury of myocardial infarction model *in vivo* and cardiomyocyte model of oxidative stress *in vitro*. Knockdown of miR-16 in AMI rats was cardioprotective, as evidenced by reduced cardiac apoptosis and infarct size, serum LDH and CK release, and improved cardiac function. Furthermore, we found that miR-16 directly targets β_2_-AR, which partially explains the mechanisms of miR-16's effects on cardiac injury. This study provides evidence that miR-16 may act as a potential therapeutic target for ischemic heart disease.

It has been well-established that miRNAs are deeply involved in the pathogenesis of cardiac injury, and interfering with the expression of certain miRNA is cardioprotective. Inhibition of miR-92a reduces infarct size and preserves cardiac function after ischemia reperfusion injury in pigs [1]. Over-expression of miR-499 inhibits cardiac apoptosis by suppressing the mitochondrial fission process [22]. Up-regulation of miR-210 under hypoxic conditions confers cardioprotection in cultured cardiomyocytes [23, 24]. Considering the large number of miRNAs, those that have been documented are just a small portion, it remains necessary to discover novel miRNAs that play key role in cardiac injury and provide more potential targets for the treatment of cardiac injury.

miR-16 located at 13q14, which is identified as miR-15 miRNA cluster that includes miR-15a, miR-15b, miR-16, miR-195, and miR-497 [25]. Studies showed that the expression of miR-16 altered in various cardiac diseases. miR-16 was up-regulated in the infarcted region 24 hours after ischemic injury in the porcine MI model [3], and down-regulated in myocardial hypertrophy of rats and mice [26]. Consistently, we also identified that the level of miR-16 was elevated in the infract area of hearts 6 h after AMI and cultured NRVCs treated with H_2_O_2_. A host of studies confirmed that miR-16 plays an important role in the regulation of cell proliferation and apoptosis [27]. Xue et al found that miR-16 aggravated cytotoxicity and apoptosis induced by taxol in breast cancer cells through suppressing IKBKB expression [28]. miR-16 also inhibited the proliferation and induced apoptosis of non-small cell lung cancer via regulating the expression of p27, Bcl-2, bax, and caspase 3 [29]. Over-expression of miR-16 significantly attenuated renal function and increased apoptosis in epithelium tubule cells in mice [30]. However, there is no experimental evidence on the effect of miR-16 on cardiac ischemia, although a member of the miR-15 family miR-15a has been proven to participate in the regulation of myocyte proliferation and apoptosis [3, 31]. In the current work, we experimentally demonstrated that miR-16 exacerbated cardiac injury by inhibiting cell viability and promoting apoptosis in cultured cardiac myocytes. Moreover, knockdown of miR-16 alleviated myocardial injury in AMI rats, as manifested by decreased infarct size, serum LDH and CK and improved cardiac function.

β_2_-AR plays a protective role in cardiac injury via different mechanisms. Alan et al reported that selective β_2_-AR stimulation protected myocytes from apoptosis induced by hypoxia or H_2_O_2_ through PI-3K survival pathway [5]. β_2_-AR activation using arformoterol attenuates myocardial cell death via NO synthase activation and causes a subsequent increase in NO bioavailability [6]. β_2_-AR also mediates the protective effect of ischemic preconditioning, which is initially coupled to the Gs, resulting in an increase in PKA activation that can phosphorylate the receptor and switch coupling to the Gi [32]. Furthermore, β_2_-AR stimulation attenuates left ventricular remodeling and decreases apoptosis in a rodent model of ischemic cardiomyopathy [33]. These works indicates the critical role of β_2_-AR in maintaining the survival of cardiac myocytes. In this study we found that the expression of β_2_-AR was markedly reduced in ischemic heart, while the expression of miR-16 was increased, indicating the potential regulation of miR-16 on β_2_-AR. Our subsequent data verified that β_2_-AR was the target gene of miR-16. Over-expression of miR-16 caused a significant reduction of β_2_-AR expression in both a cellular model of oxidative stress and a rat model of AMI. These data indicated that the detrimental effect of miR-16 on cardiac myocytes is conferred by the down-regulation of β_2_-AR.

One of miRNA's biological functions is that one miRNA can regulate the expression of multiple genes [34]. Except for β_2_-AR, a number of apoptosis related genes have been validated to be the target gene of miR-16. Chen et al. showed that miR-16 aggravated renal ischemia reperfusion injury by targeting bcl-2 [30]. Studies showed that miR-16 targets the 3′ UTR of vascular endothelial growth factor (VEGF) to regulate angiogenesis [35, 36]. In addition to bcl-2 and VEGF, TGF-β, PI3K-Akt and p53 are considered to be the potential targets of miR-16 [37]. In this study, we identified β_2_-AR as a direct target gene of miR-16, and did not investigate other potential targets. We can't rule out the possible involvement of all the validated target genes and other potential targets of miR-16 in its effects on cardiac myocytes, which is one of the limitations of our current work.

In summary, our work demonstrated that miR-16 was significantly up-regulated during cardiac ischemia, and suppression of miR-16 protects against acute myocardial infarction. Suppression of β_2_-AR expression may underlie the harmful effects of miR-16 on the heart. Our work implies that miR-16 may be potential therapeutic target for the treatment of ischemic heart disease.

## MATERIALS AND METHODS

### Animals

Healthy male Sprague Dawley rats (200 ± 20 g) were purchased from the Animal Center of the Second Affiliated Hospital of Harbin Medical University (Harbin, Heilongjiang Province, China). The experimental rats were bred in a standard condition (temperature 21±1°C, humidity 55-60%). Animals were given water and food ad libitum. All animal experimental procedures were in accordance with and approved by the ethic committee of Harbin Medical University.

### Rat model of AMI

Rat AMI model was established as previously described [38]. Briefly, rats were anesthetized by intraperitoneal injection of sodium pentobarbital (60 mg/kg). The chest was opened at the fourth intercostals space and the heart was exposed. The LAD was ligated with 5/0 silk suture. The successful establishment of AMI model was confirmed by the apparent elevation of S-T segment in electrocardiogram (ECG) and cyanosis of the myocardium. Sham rats underwent the same operation procedures without ligation of coronary artery. Rats were sacrificed 6 h after LAD ligation. Cardiac tissues were collected and stored at -80°C for subsequent experiments.

### Infarction area measurement

Infarct size was determined by Evans blue (Sigma-Aldrich, USA) and TTC (Solarbio Life Sciences, Beijing, China) staining as described previously [39]. Briefly, rats were anesthetized 6 h after LAD ligation, and Evans blue dye (5%, 0.5 ml) was injected into the inferior vena cava to identify ischemic and non-ischemic myocardium. The hearts were quickly removed and washed with normal saline. Hearts were sectioned into 2 mm thick slices from apex to base, and stained with 2% TTC solution at 37°C for 20 min. The IA was pale while the normal area was red after TTC staining. IA and AAR were calculated using Image ProPlus 5.0 software. Myocardial infarct size was evaluated by the ratio of IA to AAR.

### Cell culture experiments

Primary NRVCs were obtained by the method as described in our previous study [40]. Briefly, hearts of neonatal SD rats (1-3 days old) were quickly removed and placed in DMEM medium (Hyclone Laboratories, USA) without serum under aseptic condition. The ventricles were cut into 1 to 2 mm^3^ and were digested with 0.25% trypsin at 37°C for 5-10 min, until the ventricular blocks were digested completely. Suspension was pelleted by centrifugation at 1500 rpm for 5 min. The suspended cells were placed in incubator for 2 h. After the fibroblasts were attached to bottom of well, cell suspension was plated into 6-well plate with DMEM medium containing 10% fetal bovine serum (FBS) (Gibco, USA) and 1% penicillin (100U/ml)/streptomycin (100U/ml) (Beyotime, Jiangsu, China) at 3×10^5^ cells per well, then cultured at 37°C in humid air with 5% CO_2_. After 48 h, the culture medium was replaced and cells were used for the subsequent studies.

### Transfection procedure and H_2_O_2_ treatment

miR-16 and AMO-16 were synthesized by RiboBio (RiboBio, Guangzhou, China). A scrambled RNA was used as a NC. Transfection of synthesized miRNAs was accomplished by using X-treme GENE siRNA Transfection Reagent (Roche, USA). The mixture of transfection reagent and miRNAs was added into the NRVCs and incubated at 37°C for 6 h. Subsequently, cells were replaced with 2 ml fresh medium containing 10% FBS and cultured in the incubator until the following experiments. Cells were used for real-time PCR and western blot analysis after transfection of miR-16 (100 nM) or AMO-16 (200 nM) for 48 h. Cardiomyocyte hypoxia injury was induced by 100 μM H_2_O_2_ (Tian Da chemical reagent Co., Ltd Tianjin, China) for 12 h treatment.

### Cell viability assay

Cell viability was determined by MTT assay. NRVCs with various treatments were seeded in 96-well culture plates with 1×10^4^ cells/well, and incubated at 37°C with 5% CO_2_. Cells in each well were added with 20 μl MTT (Sigma-Aldrich St Louis, USA) solution (5 mg/ml) and incubated for 4 h. Formazan crystals were then dissolved in 150 μl dimethyl sulfoxide. After rocking for 10 min, the absorbance value of each well was measured with a microplate reader (BioTek, Richmond, USA) at 490 nm.

### TUNEL analysis

Myocardial cell apoptosis was evaluated by TUNEL method with the *In situ* Cell Death Detection Kit (Roche, Indianapolis, USA) according to the manufacturer's instructions. After TUNEL staining, myocardial cells were stained with DAPI (Biosharp, China) solution for 20 min at room temperature. The samples were observed under fluorescence microscope (Confocal microscopy Carl Zeiss LSM700).

### Serum LDH and CK measurement

Serum LDH and CK were measured using LDH and CK detection kits (Nanjing Jiancheng Bioengineering Institute, China) according to the manufacturer's instructions.

### Luciferase reporter assay

The full length of 3′UTR of β_2_-AR gene containing the predicated target sites for miR-16 was amplified by PCR amplification. The fragment was into the psi-CHECK2 luciferase reporter vector (Promega, Beijing, China). HEK-293 cells were co-transfected with the plasmids of luciferase reporters containing β_2_-AR 3′UTR and miR-16 or AMO- miR-16 or negative control using Lipofectamine 2000 (Invitrogen, Carlsbad, USA). The cell lysates were harvested 48 h after transfection and the luciferase activity was measured with a Dual-Luciferase Reporter Assay System (Promega, Beijing, China) according to manufacturer's instruction.

### *In vivo* transfection of lentivirus into rat hearts

Virus-containing solution (2×10^6^ TU in 70 μl of DMEM) including len-NC, len-pre-miR-16, or len-AMO-16 was injected into left ventricular cavity of rat heart using an insulin syringe. Animals in the control group were given the same volume of DMEM. Ligation of LAD was performed at day 7 after injection. In order to reduce postoperative infection, rats were given penicillin (1×10^5^ Units/day, im) for 7 days.

### Western blotting

Total protein was extracted with the procedures as described [41]. Briefly, Protein samples (80-100 μg) were separated by 10% acrylamide gel electrophoresis (SDS-PAGE), and then transferred to nitrocellulose membrane. The membranes were blocked with 5% defatted milk for 2-3 h at room temperature on a rocker and then incubated with primary antibodies β_2_-AR (Abcam, Cambridge, MA, UK) and beta-actin (Proteintech, Wuhan, China) at 4°C overnight. After washing with PBS-T (PBS containing 0.5% Tween 20) for 3 times, the membranes were incubated with infrared fluorescent dye-labeled secondary antibody (LI-COR Biosciences, Lincoln, USA) for 50 min at room temperature away from light. Western blot bands were acquired using Odyssey infrared scanning system (LI-COR Biosciences, Lincoln, USA) and analyzed using Image Studio Ver 4.0 software.

### Real-time PCR analysis

Total RNA samples were isolated from cultured NRVCs and cardiac tissues using trizol reagent (Invitrogen, Carlsbad, California, USA) according to manufacturer's protocol. The relative expression levels of miRNAs were quantified by the Reverse Transcription Master Kit (Toyobo, Osaka, Japan) and real-time RT-PCR with SYBR Green I (Toyobo, Osaka, Japan). cDNA was amplified in an ABI 7500 fast system (Applied Biosystems, CA, USA), using the same cycling parameters as follows: 95°C for 10 min followed by 40 cycles of a three-stage temperature profile of 95°C for 15 sec and 60°C for 15 sec, then 72°C for 30 sec. Sequences of gene-specific PCR primers were used as follows: miR-16: RT: 5′- GTCGTATCCAGTGCGTGTCGTGGAGTCGGCAATTGCACTGGATACGACCGCCAAT-3′. Forward: 5′- GGGGTAGCAGCACGTAAAT -3′, Reverse: 5′- TGTCGTGGAGTCGGCAATTG -3′, U6: Forward: 5′- GCTT CGGC AGCA CATA TACT AAAA T-3′, Reverse: 5′- CGCT TCAC GAAT TTGC GTGT CAT-3′. U6 was used as an internal control for measurement.

### Echocardiographic measurement

Rats in treatment group received len-pre-miR-16, len-AMO-16 or len-NC. Ligation of LAD was performed in rats 7 days after the above treatment. Cardiac function was evaluated in rats 6 h after AMI with transthoracic echocardiography using an ultrasound machine Vevo2100 high-resolution imaging system (Visual Sonics, Toronto, ON, Canada) equipped with a 10-MHz phased-array transducer, and heart function evaluation in control group was performed 6 h after LAD ligation. Rats were sedated with 3% sodium pentobarbital. Ejection factor, fractional shortening, and cardiac parameters were calculated from M-mode recording.

### Statistical analysis

Data are expressed as mean ± SD. Statistical analysis was performed using Student's test. A two-tailed P < 0.05 was considered as statistically difference.

## Abbreviations

β_2_-AR: beta2-adrenoreceptor
NRVCs: neonatal rat ventricular cells
β_1_-AR: beta1-adrenoreceptor
cAMP: cyclic adenosine monophosphate
H_2_O_2_: hydrogen peroxide
LAD: left anterior descending coronary artery
AMO: antisense oligodeoxyribonucleotide
NC: negative control
MTT: 3-[4,5-dimethylthiazol -2-yl]-2, 5 diphenyl tetrazolium bromide
TTC: triphenyltetrazolium chloride
IA: infarct area
AAR: area at risk
LDH: lactate dehydrogenase
CK: creatine kinase
EF: ejection fraction
FS: fractional shortening
VEGF: vascular endothelial growth factor
ECG: electrocardiogram
FBS: fetal bovine serum.

## References

[1] Hinkel R, Penzkofer D, Zuhlke S, Fischer A, Husada W, Xu QF, Baloch E, van Rooij E, Zeiher AM, Kupatt C, Dimmeler S (2013). Inhibition of microRNA-92a protects against ischemia/reperfusion injury in a large-animal model. Circulation.

[2] Gerczuk PZ, Kloner RA (2012). An update on cardioprotection: a review of the latest adjunctive therapies to limit myocardial infarction size in clinical trials. Journal of the American College of Cardiology.

[3] Hullinger TG, Montgomery RL, Seto AG, Dickinson BA, Semus HM, Lynch JM, Dalby CM, Robinson K, Stack C, Latimer PA, Hare JM, Olson EN, van Rooij E (2012). Inhibition of miR-15 protects against cardiac ischemic injury. Circulation research.

[4] Communal C, Singh K, Sawyer DB, Colucci WS (1999). Opposing effects of beta(1)- and beta(2)-adrenergic receptors on cardiac myocyte apoptosis: role of a pertussis toxin-sensitive G protein. Circulation.

[5] Chesley A, Lundberg MS, Asai T, Xiao RP, Ohtani S, Lakatta EG, Crow MT (2000). The beta(2)-adrenergic receptor delivers an antiapoptotic signal to cardiac myocytes through G(i)-dependent coupling to phosphatidylinositol 3'-kinase. Circulation research.

[6] Bhushan S, Kondo K, Predmore BL, Zlatopolsky M, King AL, Pearce C, Huang H, Tao YX, Condit ME, Lefer DJ (2012). Selective beta2-adrenoreceptor stimulation attenuates myocardial cell death and preserves cardiac function after ischemia-reperfusion injury. Arteriosclerosis, thrombosis, and vascular biology.

[7] Fajardo G, Zhao M, Berry G, Wong LJ, Mochly-Rosen D, Bernstein D (2011). beta2-adrenergic receptors mediate cardioprotection through crosstalk with mitochondrial cell death pathways. Journal of molecular and cellular cardiology.

[8] Patterson AJ, Zhu W, Chow A, Agrawal R, Kosek J, Xiao RP, Kobilka B (2004). Protecting the myocardium: a role for the beta2 adrenergic receptor in the heart. Critical care medicine.

[9] Ahmet I, Lakatta EG, Talan MI (2005). Pharmacological stimulation of beta2-adrenergic receptors (beta2AR) enhances therapeutic effectiveness of beta1AR blockade in rodent dilated ischemic cardiomyopathy. Heart failure reviews.

[10] Tsuneyoshi H, Oriyanhan W, Kanemitsu H, Shiina R, Nishina T, Matsuoka S, Ikeda T, Komeda M (2005). Does the beta2-agonist clenbuterol help to maintain myocardial potential to recover during mechanical unloading?. Circulation.

[11] Bartel DP (2004). MicroRNAs: genomics, biogenesis, mechanism, and function. Cell.

[12] van Rooij E, Olson EN (2007). MicroRNAs: powerful new regulators of heart disease and provocative therapeutic targets. The Journal of clinical investigation.

[13] Latronico MV, Catalucci D, Condorelli G (2007). Emerging role of microRNAs in cardiovascular biology. Circulation research.

[14] Callis TE, Wang DZ (2008). Taking microRNAs to heart. Trends in molecular medicine.

[15] Ren XP, Wu J, Wang X, Sartor MA, Qian J, Jones K, Nicolaou P, Pritchard TJ, Fan GC (2009). MicroRNA-320 is involved in the regulation of cardiac ischemia/reperfusion injury by targeting heat-shock protein 20. Circulation.

[16] Hu S, Huang M, Li Z, Jia F, Ghosh Z, Lijkwan MA, Fasanaro P, Sun N, Wang X, Martelli F, Robbins RC, Wu JC (2010). MicroRNA-210 as a novel therapy for treatment of ischemic heart disease. Circulation.

[17] Liu Z, Ye P, Wang S, Wu J, Sun Y, Zhang A, Ren L, Cheng C, Huang X, Wang K, Deng P, Wu C, Yue Z (2015). MicroRNA-150 protects the heart from injury by inhibiting monocyte accumulation in a mouse model of acute myocardial infarction. Circulation Cardiovascular genetics.

[18] Bonci D, Coppola V, Musumeci M, Addario A, Giuffrida R, Memeo L, D'Urso L, Pagliuca A, Biffoni M, Labbaye C, Bartucci M, Muto G, Peschle C (2008). The miR-15a-miR-16-1 cluster controls prostate cancer by targeting multiple oncogenic activities. Nature medicine.

[19] Rivas MA, Venturutti L, Huang YW, Schillaci R, Huang TH, Elizalde PV (2012). Downregulation of the tumor-suppressor miR-16 via progestin-mediated oncogenic signaling contributes to breast cancer development. Breast cancer research: BCR.

[20] Linsley PS, Schelter J, Burchard J, Kibukawa M, Martin MM, Bartz SR, Johnson JM, Cummins JM, Raymond CK, Dai H, Chau N, Cleary M, Jackson AL (2007). Transcripts targeted by the microRNA-16 family cooperatively regulate cell cycle progression. Molecular and cellular biology.

[21] Duisters RF, Tijsen AJ, Schroen B, Leenders JJ, Lentink V, van der Made I, Herias V, van Leeuwen RE, Schellings MW, Barenbrug P, Maessen JG, Heymans S, Pinto YM (2009). miR-133 and miR-30 regulate connective tissue growth factor: implications for a role of microRNAs in myocardial matrix remodeling. Circulation research.

[22] Wang JX, Jiao JQ, Li Q, Long B, Wang K, Liu JP, Li YR, Li PF (2011). miR-499 regulates mitochondrial dynamics by targeting calcineurin and dynamin-related protein-1. Nat Med.

[23] Mutharasan RK, Nagpal V, Ichikawa Y, Ardehali H (2011). microRNA-210 is upregulated in hypoxic cardiomyocytes through Akt- and p53-dependent pathways and exerts cytoprotective effects. American journal of physiology Heart and circulatory physiology.

[24] Chen M, Ma G, Yue Y, Wei Y, Li Q, Tong Z, Zhang L, Miao G, Zhang J (2014). Downregulation of the miR-30 family microRNAs contributes to endoplasmic reticulum stress in cardiac muscle and vascular smooth muscle cells. International journal of cardiology.

[25] Calin GA, Dumitru CD, Shimizu M, Bichi R, Zupo S, Noch E, Aldler H, Rattan S, Keating M, Rai K, Rassenti L, Kipps T, Negrini M (2002). Frequent deletions and down-regulation of micro- RNA genes miR15 and miR16 at 13q14 in chronic lymphocytic leukemia. Proceedings of the National Academy of Sciences of the United States of America.

[26] Huang S, Zou X, Zhu JN, Fu YH, Lin QX, Liang YY, Deng CY, Kuang SJ, Zhang MZ, Liao YL, Zheng XL, Yu XY, Shan ZX (2015). Attenuation of microRNA-16 derepresses the cyclins D1, D2 and E1 to provoke cardiomyocyte hypertrophy. Journal of cellular and molecular medicine.

[27] Aqeilan RI, Calin GA, Croce CM (2010). miR-15a and miR-16-1 in cancer: discovery, function and future perspectives. Cell death and differentiation.

[28] Tang X, Jin L, Cao P, Cao K, Huang C, Luo Y, Ma J, Shen S, Tan M, Li X, Zhou M (2016). MicroRNA-16 sensitizes breast cancer cells to paclitaxel through suppression of IKBKB expression. Oncotarget.

[29] Wang W, Chen J, Dai J, Zhang B, Wang F, Sun Y (2016). MicroRNA-16-1 Inhibits Tumor Cell Proliferation and Induces Apoptosis in A549 Non-Small Cell Lung Carcinoma Cells. Oncology research.

[30] Chen HH, Lan YF, Li HF, Cheng CF, Lai PF, Li WH, Lin H (2016). Urinary miR-16 transactivated by C/EBPbeta reduces kidney function after ischemia/reperfusion-induced injury. Scientific reports.

[31] Porrello ER, Mahmoud AI, Simpson E, Johnson BA, Grinsfelder D, Canseco D, Mammen PP, Rothermel BA, Olson EN, Sadek HA (2013). Regulation of neonatal and adult mammalian heart regeneration by the miR-15 family. Proceedings of the National Academy of Sciences of the United States of America.

[32] Tong H, Bernstein D, Murphy E, Steenbergen C (2005). The role of beta-adrenergic receptor signaling in cardioprotection. FASEB journal: official publication of the Federation of American Societies for Experimental Biology.

[33] Xydas S, Kherani AR, Chang JS, Klotz S, Hay I, Mutrie CJ, Moss GW, Gu A, Schulman AR, Gao D, Hu D, Wu EX, Wei C (2006). beta(2)-Adrenergic stimulation attenuates left ventricular remodeling, decreases apoptosis, and improves calcium homeostasis in a rodent model of ischemic cardiomyopathy. The Journal of pharmacology and experimental therapeutics.

[34] Ambros V (2001). microRNAs: tiny regulators with great potential. Cell.

[35] Hua Z, Lv Q, Ye W, Wong CK, Cai G, Gu D, Ji Y, Zhao C, Wang J, Yang BB, Zhang Y (2006). MiRNA-directed regulation of VEGF and other angiogenic factors under hypoxia. PloS one.

[36] Ye W, Lv Q, Wong CK, Hu S, Fu C, Hua Z, Cai G, Li G, Yang BB, Zhang Y (2008). The effect of central loops in miRNA: MRE duplexes on the efficiency of miRNA-mediated gene regulation. PloS one.

[37] Pan Q, Guo C, Sun C, Fan J, Fang C (2014). Integrative analysis of the transcriptome and targetome identifies the regulatory network of miR-16: an inhibitory role against the activation of hepatic stellate cells. Bio-medical materials and engineering.

[38] Li X, Wang B, Cui H, Du Y, Song Y, Yang L, Zhang Q, Sun F, Luo D, Xu C, Chu W, Lu Y, Yang B (2014). let-7e replacement yields potent anti-arrhythmic efficacy via targeting beta 1-adrenergic receptor in rat heart. Journal of cellular and molecular medicine.

[39] Pan Z, Sun X, Ren J, Li X, Gao X, Lu C, Zhang Y, Sun H, Wang Y, Wang H, Wang J, Xie L, Lu Y, Yang B (2012). miR-1 exacerbates cardiac ischemia-reperfusion injury in mouse models. PloS one.

[40] Benzhi C, Limei Z, Ning W, Jiaqi L, Songling Z, Fanyu M, Hongyu Z, Yanjie L, Jing A, Baofeng Y (2009). Bone marrow mesenchymal stem cells upregulate transient outward potassium currents in postnatal rat ventricular myocytes. Journal of molecular and cellular cardiology.

[41] Yang B, Lin H, Xiao J, Lu Y, Luo X, Li B, Zhang Y, Xu C, Bai Y, Wang H, Chen G, Wang Z (2007). The muscle-specific microRNA miR-1 regulates cardiac arrhythmogenic potential by targeting GJA1 and KCNJ2. Nature medicine.

